# Salicylic Acid Alleviates the Adverse Effects of Salt Stress in *Torreya grandis* cv. Merrillii Seedlings by Activating Photosynthesis and Enhancing Antioxidant Systems

**DOI:** 10.1371/journal.pone.0109492

**Published:** 2014-10-10

**Authors:** Tingting Li, Yuanyuan Hu, Xuhua Du, Hui Tang, Chaohua Shen, Jiasheng Wu

**Affiliations:** 1 Nurturing Station for the State Key Laboratory of Subtropical Silviculture, Zhejiang A & F University, Lin’an, Hangzhou, P. R. China; 2 School of Forestry & Biotechnology, Zhejiang A & F University, Lin’an, Hangzhou, P. R. China; 3 China National Bamboo Research Center, Hangzhou, P. R. China; Institute of Genetics and Developmental Biology, Chinese Academy of Sciences, China

## Abstract

**Background:**

Salt stress is a major factor limiting plant growth and productivity. Salicylic acid (SA) has been shown to ameliorate the adverse effects of environmental stress on plants. To investigate the protective role of SA in ameliorating salt stress on *Torreya grandis* (*T. grandis*) trees, a pot experiment was conducted to analyze the biomass, relative water content (RWC), chlorophyll content, net photosynthesis (Pn), gas exchange parameters, relative leakage conductivity (REC), malondialdehyde (MDA) content, and activities of superoxide dismutase (SOD) and peroxidase (POD) of *T. grandis* under 0.2% and 0.4% NaCl conditions with and without SA.

**Methodology/Principal Findings:**

The exposure of *T. grandis* seedlings to salt conditions resulted in reduced growth rates, which were associated with decreases in RWC and Pn and increases in REC and MDA content. The foliar application of SA effectively increased the chlorophyll (chl (a+b)) content, RWC, net CO_2_ assimilation rates (Pn), and proline content, enhanced the activities of SOD, CAT and POD, and minimized the increases in the REC and MDA content. These changes increased the capacity of *T. grandis* in acclimating to salt stress and thus increased the shoot and root dry matter. However, when the plants were under 0% and 0.2% NaCl stress, the dry mass of the shoots and roots did not differ significantly between SA-treated plants and control plants.

**Conclusions:**

SA induced the salt tolerance and increased the biomass of *T. grandis* cv. by enhancing the chlorophyll content and activity of antioxidative enzymes, activating the photosynthetic process, and alleviating membrane injury. A better understanding about the effect of salt stress in *T. grandis* is vital, in order gain knowledge over expanding the plantations to various regions and also for the recovery of *T. grandis* species in the future.

## Introduction

Salt stress is one of the major abiotic stress factors that limit plant growth and productivity [1]. Salt stress alters various biochemical and physiological responses in plants, thereby affecting almost all plant processes [2]. The major effects of salinity are significant reductions in growth, such as decreased leaf area, leaf length and root and shoot dry weight [3]. High salt stress can also restrict photosynthesis by decreasing the abundance of green pigments and causing stomatal closure and oxidative stress, thereby resulting in the formation of reactive oxygen species (ROS) [4], [5], [6]. Excessive amounts of ROS can enhance membrane lipid peroxidation, cause electrolyte leakage, and damage the chloroplast, thus inhibiting photochemical reactions and decreasing photosynthesis [7], [8]. To alleviate the harmful effects of these ROS, plants have evolved an effective scavenging system composed of non-enzymatic antioxidants (carotenoids, ascorbate, and tocopherol) and enzymatic antioxidants, such as catalase (CAT) and peroxidase (POD). Gossett et al. [9] reported that treatment with 150 mM NaCl resulted in a 72% increase in the POD activity in salt-tolerant cotton. Therefore, it is necessary to improve the salinity tolerance of plants and exploit various compounds to alleviate plant stress.

As an important signaling molecule, salicylic acid (SA) influences various physiological and biochemical functions in plants and has diverse effects on the tolerance to biotic and abiotic stress [10], [11], [12]. The role of SA in plant tolerance to abiotic stresses such as ozone, UV-B, heat, heavy metal, and osmotic stress has been reported [12], [13]. Khan et al. [14] reported that SA application alleviated the adverse effects of salt stress in mungbean through the improvement of plant photosynthesis and growth and also enhancing antioxidant system. However, the improvement of salt tolerance by exogenous SA depends on genotype and the concentration of SA used. For example, Arfan et al. [10] reported that exogenous SA counteracted the salt stress-induced growth inhibition in a salt-tolerant wheat genotype, although no improvement occurred in a salt-sensitive cultivar. Therefore, the questions of whether the effectiveness of SA in the alleviation of salt stress is dependent on the plant species must be further elucidated.

The genus Torreya is an endangered and primitive member of the yew family (Taxaceae) and consists only of six species with a restricted worldwide distribution [15]. The conservation and recovery of these species have received increasing attention in recent years. *Torreya grandis* (*T. grandis*) cv. Merrillii, belonging to the genus Torreya family Taxaceae, is an important economic tree species and has drupe-like fruits with nut seeds that were used as food for thousands years in China. *T. grandis* has also important medicinal value due to its anthelmintic, antitussive, carminative, laxative, antifungal, antibacterial, and antitumor activity [16]. Currently, *T. grandis* is only planted on hills and mountain slopes in south China. The expansion of *T. grandis* plantations on new sites is important for the recovery of *T. grandis* species. However, throughout the entire country, 99 million hm^2^ arable land is adversely affected by high salt concentrations, and the soil salinization is forecasted to increase drastically in the coming decades due to the worldwide ongoing climate change [17]. Therefore, it is necessary to improve the salinity tolerance of *T. grandis* species to expand its plantation under stress conditions. For the past years, numerous studies have been focused over *T. grandis* app:addword:concentratespecies for their ecological characterization; chemical constituents, fatty acid composition and its distribution in the seeds and the therapeutic use of the seed extracts [18], [19], [20], [21]. Unfortunately, few published studies are available on the effect of salt stress on the growth and physiology of *T. grandis* trees. Additionally, it remains unclear whether SA plays a significant protective role in ameliorating the influence of salt stress on *T. grandis* trees to increase its salt tolerance.

The objective of this study was to investigate the effects of exogenous SA application on *T. grandis* growth and physiology under 0.2% and 0.4% NaCl conditions by analyzing the biomass, photosynthesis, membrane injury, and antioxidative enzyme activity. The information obtained is essential for propagation and cultivation of T. grandis and the other Torreya species under salt stress condition.

## Materials and Methods

### Plant materials and growth conditions

The pot experiments under different salt stress treatments were conducted in a controlled environment room of the College of Forest and Biotechnology, Zhejiang Agriculture and Forestry University, Zhejiang Province, China (N30°23′, E119°72′) in 2012. In March, two-year-old healthy and homogenous *T. grandis* seedlings were transplanted from Chunan, Zhejiang Province, China, and transferred to plastic pots (13.5 cm inner-diameter and 16 cm height with holes in the bottom, one seedling per pot) that were filled with 4 kg mixture of silt and perlite soil (3∶1, v/v, pH 6.40). The soil was a loam with an organic matter content of 2.90%, total N content of 0.255%, total P content of 0.125%, and total K content of 1.574%. Four weeks after transplanting, a completely randomized design with 3 replications per treatment and 5 plants per replication was adopted. Six treatments with different salt concentrations (0, 0.2 and 0.4% NaCl) and different SA concentrations (0 and 0.5 mmol) were implemented as follows: T1 (distilled water without SA); T2 (distilled water with 0.5 mmol SA); T3 (0.2% NaCl without 0.5 mmol SA); T4 (0.2% NaCl with 0.5 mmol SA); T5 (0.4% NaCl without 0.5 mmol SA); and T6 (0.4% NaCl with 0.5 mmol SA). To avoid osmotic shock, the NaCl solution was gradually added to the soil in eight steps to achieve the final concentrations of 0.2% and 0.4% in 4 days. SA was dissolved in ethanol and then added to “Tween-20” (0.1% dilute solution) to facilitate spreading of the solution on the plant-leaf surface. The desired SA concentration was attained using double-distilled water (DDW). SA was sprayed over the leaves 3 days before NaCl treatment and also continued during the NaCl application. SA was sprayed over the adaxial and abaxial surfaces of a leaf until dripping a common insecticidal atomizer and it was done for twice a day at the time interval of 07:00 and18:00. When SA solution was sprayed, the surface of the potting soil was covered with plastic film in case SA solution dripped to the root. The plants were then collected for biomass analysis after 60 days, and measurements of other indexes were collected after 30 days of treatments. Twice in week irrigation were done for different treatmental plants with their corresponding solution in order to maintain the field capacity at 70–75%. The environmental conditions were maintained as same throughout the experiment.

### Analysis of growth and biomass

Sixty days after the treatments, one intact plant from each replicate of treatments was uprooted for biomass analysis. The green plant tissues and roots were oven-dried at 65°C to a constant weight and weighed using an electronic scale to determine the biomass.

### Determination of the leaf relative water content (RWC) and proline content

A total of 0.5 g of fresh leaf tissue was immediately weighed (fresh weight, WF) after rehydrating for 24 h in the dark (saturated weight, WS) and after oven-drying at 85°C for 24 h to a constant weight (WD). The RWC was calculated using the following formula:



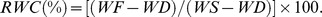



Proline accumulation was determined as described by Bates et al. [22] with slight modifications. Approximately 0.2 g of fresh leaf material was homogenized in 5 mL 3% aqueous sulfosalicylic acid. The samples were mixed, heated in boiling water for 30 min, and filtered through Whatman's No. 2 filter paper. A total of 2 mL of filtrate was mixed with 2 mL of acid-ninhydrin and 2 mL of glacial acetic acid in a test tube. The mixture was placed in a water bath for 1 h at 100°C and cooled to room temperature. The reaction mixture was extracted with 5 mL of toluene, and the chromophore containing toluene was aspirated. The absorbance was measured at 520 nm using toluene as a blank. The proline concentration was calculated using proline standards (0–50 mg/mL).

### Analysis of gas exchange

The youngest healthy, fully developed leaves from a randomly selected branch were used for the gas exchange measurements on recently expanded leaves using a portable photosynthesis measuring instrument (LI-6400, LiCor, Inc. Lincoln, NE, USA) equipped with an 6400-05 conifer chamber at concentration of 400 µmol mol^−1^ CO_2_, 21% O_2_, and 50% relative humidity with a natural light source. The temperature of the chamber was maintained at 29–32°C. Photosynthetic gas exchange measurements were made during 9:00–10:30 when the light intensity was between 1200–1600 µmol m^−2^s^−1^. This irradiance was higher than the saturation irradiance for current year leaf of *T. grandis* (452–514 µmol m^−2^ s^−1^, data not shown). Therefore, light was not the limitation for photosynthesis in these gas exchange measurements.

### Determinations of the chlorophyll concentration and soluble protein content

A total of 0.1 g of leaf discs was weighed, placed into a test tube, and then repeatedly extracted with 8 mL of 95% (V/V) ethanol. The photosynthetic pigments were extracted at 4°C for 48 h in the darkness. The absorbance of the supernatant was measured at 646 and 663 nm. The chlorophyll a (Chla) and Chlb concentration was determined according to the methods of Lichtenthaler [23].

The soluble protein content was measured according to the procedure of Bradford [24] with a slight change. A 0.5-g frozen sample was extracted in sodium phosphate buffer (50 mmol, pH 7.8). The extracts were centrifuged at 4000 ×*g* for 10 min at 4°C. The soluble proteins were quantified using the supernatants of samples with bovine serum albumin as a standard.

### Determinations of the relative electrolyte leakage rate and lipid peroxidation

To measure the electrolyte leakage, 10 leaf discs (10 mm diameter) from young, fully expanded leaves were placed in 50-mL glass vials and rinsed with distilled water to remove electrolytes that were released during the leaf disc excision. The vials were then filled with 30 mL of distilled water and allowed to stand in the dark for 24 h at room temperature. The electrical conductivity (EC_1_) of the bathing solution was determined at the end of incubation period. The vials were heated in a temperature-controlled water bath at 95°C for 20 min and then cooled to room temperature, after which the electrical conductivity (EC_2_) was measured. The relative electrolyte leakage (REC) was calculated using the following formula [25]:



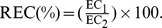



The degree of lipid peroxidation was assessed based on the malondialdehyde (MDA) contents according to Zheng et al. [26]. A volume of 2 mL of the supernatant sample was combined with 2 mL of thiobarbituric acid (TBA) incubated in boiling water for 30 min and then quickly cooled in an ice bath. The mixture was centrifuged at 5000 × g for 10 min, and the absorbance of supernatant was monitored at 532, 600 and 450 nm. The MDA concentration was expressed as μmol g^−1^ using the following formula:



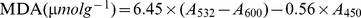
.

### Determination of antioxidative enzyme activities

The enzymes were extracted at 4°C from approximately 0.3 g of tissue from the second leaves using a mortar and pestle with 5 mL of extraction buffer, containing 0.1 mol phosphate buffer (pH 7.8), 0.1 mol ethylenediaminetetraacetic acid (EDTA), and 1% polyvinylpyrrolidone (PVP). The extracts were centrifuged at 6000 × g for 30 min, and the supernatants were used for the enzyme assays.

The superoxide dismutase (SOD) activity was assayed by the inhibition of the photochemical reduction of β-nitroblue tetrazolium chloride (NBT). The reaction mixture (3 mL) consisted of 50 mol potassium phosphate buffer (pH 7.8) containing 0.3 µmol EDTA, 39.15 µmol methionine, 0.225 µmol nitroblue tetrazolium, 0.006 µmol riboflavin, and 0.05 mL of enzyme extract. The reaction was conducted for 15 min at 25°C under 4000 l×. One unit of SOD activity was defined as the amount of enzyme that inhibited the NBT reduction by 50% at 560 nm [27].

The CAT activity was assayed by monitoring the disappearance of hydrogen peroxide (H_2_O_2_) [28]. This disappearance can be detected by measuring the decrease in the absorbance at 240 nm of a reaction mixture consisting of 1.5 mL of 50 mmol sodium phosphate buffer (pH 7.8), 0.3 mL of 100 mmol H_2_O_2_, and 0.2 mL of enzyme extract. One CAT unit was defined as the amount of enzyme that was necessary to decompose 1 mmol H_2_O_2_ per minute under the aforementioned assay conditions, and the specific activity was expressed as U g^−1^ FW min^−1^.

The POD activity was determined based on guaiacol oxidation measured at 470 nm [29]. The reaction mixture (3 mL) contained 0.05 mL of enzyme extract, 2.75 mL of 50 mmol phosphate buffer (pH 7.0), 0.1 mL of 1% H_2_O_2_, and 0.1 mL of a 4% guaiacol solution. The increase in absorbance at 470 nm due to guaiacol oxidation was recorded for 2 min. One unit of enzyme activity was defined as the amount of the enzyme that caused a change of 0.01 absorbance unit per minute. The specific POD activity was expressed as U g^−1^ FW min^−1^.

### Statistical analyses

The experiment was conducted according to a simple randomized block design. Each treatment was replicated three times. The data were statistically analyzed by an analysis of variance (ANOVA) using SAS 9.2 Institute Inc. (2008) software. The least significant difference (LSD) was calculated to separate the means. A simple correlation analysis was performed using the SAS program to determine the relationship between each physiological variable.

## Results

### Dry mass of the shoots and roots of *T. grandis* under different salt stress and SA treatments

**Figure 1:**
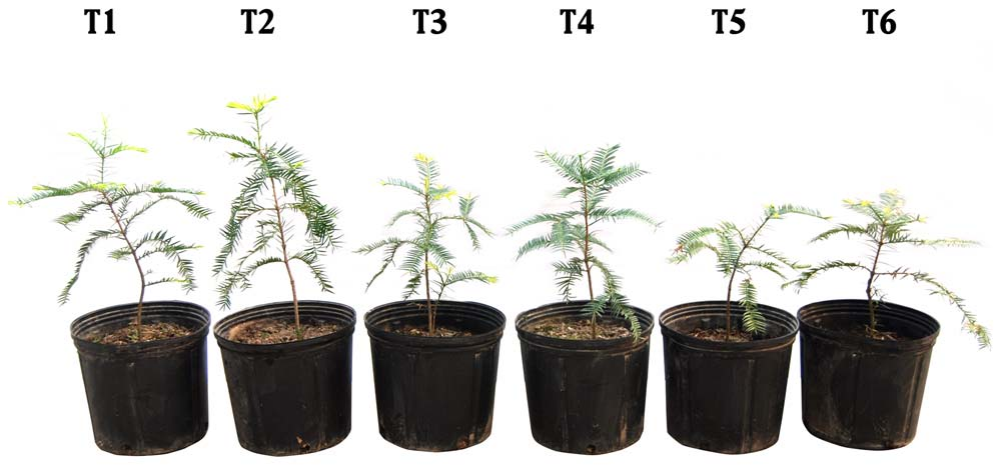
The appearance of whole plants in *T. grandis* seedlings. Treatments: T1, distilled water without SA; T2, distilled water with 0.5 mmol SA; T3, 0.2% NaCl without SA; T4, 0.2% NaCl with 0.5 mmol SA; T5, 0.4% NaCl without SA; and T6, 0.4% NaCl with 0.5 mmol SA.

Salt stress significantly inhibited the plant growth, leading to a pronounced reduction in the dry mass of the shoots and roots. Compared with the non-salt-treated *T. grandis* seedlings (T1), the 0.2% and 0.4% NaCl treatments significantly reduced the dry mass by 12.1% and 29% in the shoots and by 9.9% and 25.3% in the roots after 60 days, respectively (Table 1). The SA treatment reduced the decrease in the growth of the salt-stressed plants. Compared with the salt-stressed plants, SA treatment increased the dry mass of the shoots, roots, and shoot+roots by 6.2% (P<0.05), 5.6% (P<0.05) and 5.9% (P<0.05) under 0.2% NaCl conditons, and 16.8% (P<0.05), 18.2% (P<0.05), and 17.7% (P<0.05) under 0.4% NaCl conditions, respectively (Table 1). However, there were no significant differences in the dry mass of shoots and roots between different treatments with or without SA treatment under 0% NaCl stress condition. In addition, the salt-stressed plants grown under SA were larger than those without SA (Fig. 1).

**Table 1:** Effects of SA on the dry matter of the shoots, roots, and roots+shoots in *T. grandis* grown under salt stress (means±SD).

Treatments	Shoots (g)	Roots (g)	Shoots+Roots (g)
T1	18.4±1.15^a^	11.9±0.68^a^	30.3±0.49^a^
T2	18.7±0.93^a^	12.0±0.39^a^	30.7±0.98^a^
T3	16.2±1.17^b^	10.7±0.37^b^	26.9±1.54^bc^
T4	17.2±1.31^ab^	11.3±0.74^ab^	28.5±1.62^ab^
T5	13.1±0.92^c^	8.88±0.56^c^	22.0±1.47^d^
T6	15.3±1.34^b^	10.5±0.45^b^	25.9±1.10^c^

Treatments: T1, distilled water without SA; T2, distilled water with 0.5 mmol SA; T3, 0.2% NaCl without SA; T4, 0.2% NaCl with 0.5 mmol SA; T5, 0.4% NaCl without SA; and T6, 0.4% NaCl with 0.5 mmol SA. Numbers followed by different letters indicate significant differences (*P*<0.05) according to an LSD test; the same letter indicates no significant differences between the treatments, n = 5.

### Relative water content and proline content of *T. grandis* under different salt stress and SA treatments

**Figure 2:**
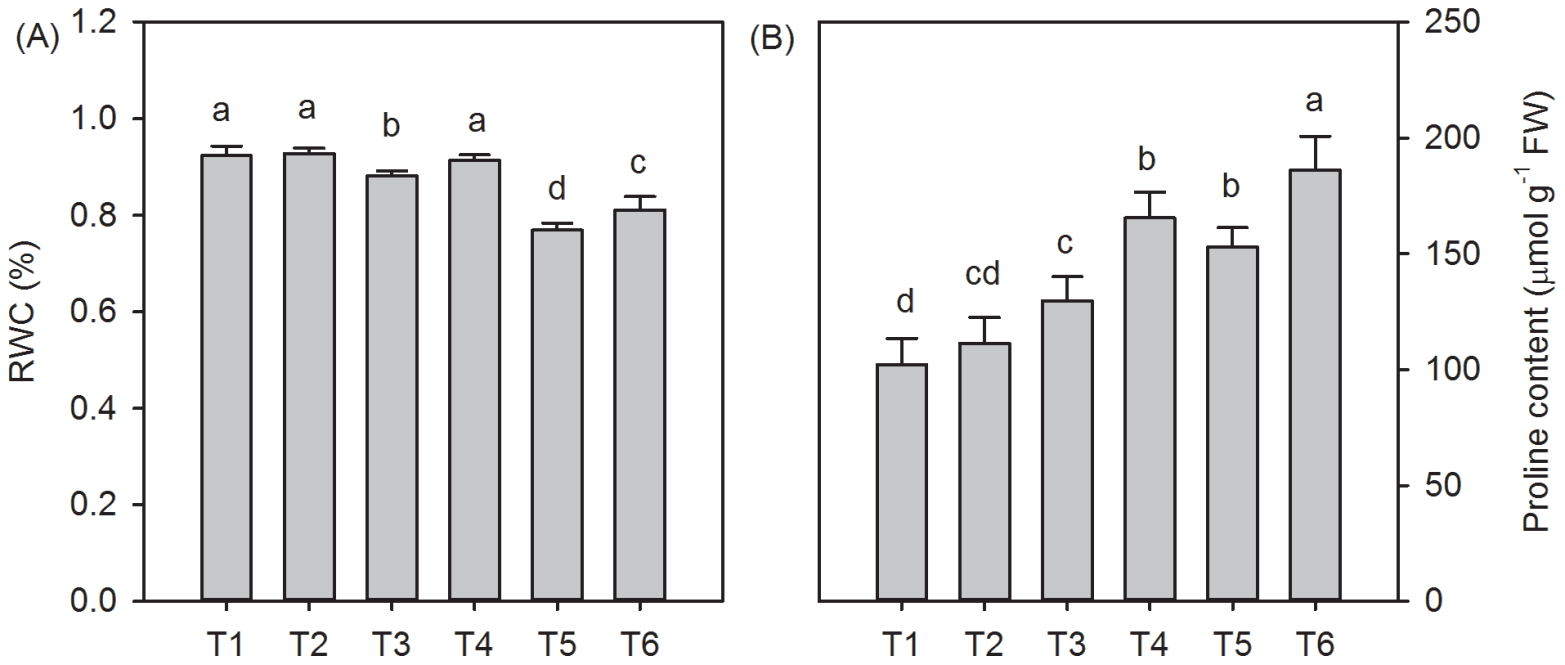
Effects of SA on the RWC (A) and proline content (B) in *T. grandis* grown under salt stress conditions (means ± SD). Treatments: T1, distilled water without SA; T2, distilled water with 0.5 mmol SA; T3, 0.2% NaCl without SA; T4, 0.2% NaCl with 0.5 mmol SA; T5, 0.4% NaCl without SA; and T6, 0.4% NaCl with 0.5 mmol SA. Different letters indicate significant differences (*P*<0.05) according to an LSD test; the same letter indicates no significant differences between the treatments, n = 5.

The RWC is a useful parameter when evaluating the physiological water status of plants [24]. Compared to the distilled water-treated seedlings (T1), the RWC in the *T. grandis* seedling leaves decreased by 4.50% (*P*<0.05) under 0.2% salt stress conditions and 16.70% (*P*<0.05) under 0.4% salt stress conditions. The 30-day treatment with 0.5 mmol SA reduced the effects of salt stress on the relative water content decrease in the presence of 0% and 0.2% NaCl; however, these changes were not significant (*P*>0.05). Under 0.4% NaCl stress conditions, the plants that were treated with SA had a higher RWC than those without SA treatment (Fig. 2A, *P*<0.05). The salt stress dramatically induced the accumulation of proline in leaves. SA treatment further increased the proline content. In the SA treatments, the proline content in the 0.2% and 0.4% salt-stressed plants increased by 27.6% (*P*<0.05) and 21.8% (*P*<0.05), respectively (Fig. 2B).

### Gas exchange parameters in *T. grandis* under different salt and SA treatments

As shown in Table 2, salt stress reduced the gas exchange parameters, with decreases in the net assimilation rate (Pn), stomatal conductance (Gs), and transpiration rate (Tr) of the leaves of 16.2% (*P*<0.05), 50% (*P*<0.05), and 30.2% (*P*<0.05), respectively, under 0.2% NaCl conditions and 65.0% (*P*<0.05), 50% (*P*<0.05), and 59.3% (*P*<0.05) under 0.4% NaCl condition, respectively. Interestingly, the intercellular CO_2_ (Ci) did not change significantly with 0.2% NaCl (*P*>0.05) but was markedly increased with 0.4% NaCl (*P*<0.05). The SA treatment increased the Pn, Tr, and Gs in leaves under salt stress. The Pn, Tr, and Gs in SA-treated plants increased by approximately 65%, 25%, and 100%, respectively, under 0.2% NaCl conditions and 146%, 95%, and 100%, respectively, under 0.4% NaCl conditions compared with the non SA-treated plants. The Ci in the SA-treated plants under 0.2% NaCl conditions was similar to that in the non SA-treated plants (*P*>0.05). However, the Ci in the SA-treated plants under 0.4% NaCl stress was markedly lower (22%) than that of the plants without SA treatment (*P*<0.05). Additionally, there were no significant differences in the Pn, Tr, Gs, and Ci values between the SA- and distilled water-treated plants.

**Table 2:** Effects of SA treatments on the net photosynthetic rate (Pn), internal carbon dioxide concentration (Ci), transpiration rate (Tr), and stomatal conductance (Gs) in *T. grandis* grown under salt stress conditions.

Treatments	Pn (μmol m^−2^s^−1^)	Ci (μmol mol^−1^)	Tr (μmol m^−2^s^−1^)	Gs (μmol m^−2^s^−1^)
T1	1.44±0.14^bc^	251.8±15.8^ab^	1.37±0.098^a^	0.02±0.002^a^
T2	1.50±0.11^b^	257.5±10.7^ab^	1.43±0.086^a^	0.02±0.016^a^
T3	1.20±0.12^d^	230.5±25.4^bc^	0.96±0.068^c^	0.01±0.001^c^
T4	1.98±0.12^a^	243.0±16.0^bc^	1.20±0.129^b^	0.02±0.001^a^
T5	0.50±0.05^e^	276.2±22.5^a^	0.56±0.035^d^	0.01±0.001^c^
T6	1.23±0.15^cd^	214.7±14.7^c^	1.09±0.108^bc^	0.02±0.002^b^

Treatments: T1, distilled water without SA; T2, distilled water with 0.5 mmol SA; T3, 0.2% NaCl without SA; T4, 0.2% NaCl with 0.5 mmol SA; T5, 0.4% NaCl without SA; and T6, 0.4% NaCl with 0.5 mmol SA. Numbers followed by different letters indicate significant differences (*P*<0.05) according to an LSD test, n = 5.

### Contents of chlorophyll and soluble protein of *T. grandis* under different salt stress and SA treatments

**Figure 3:**
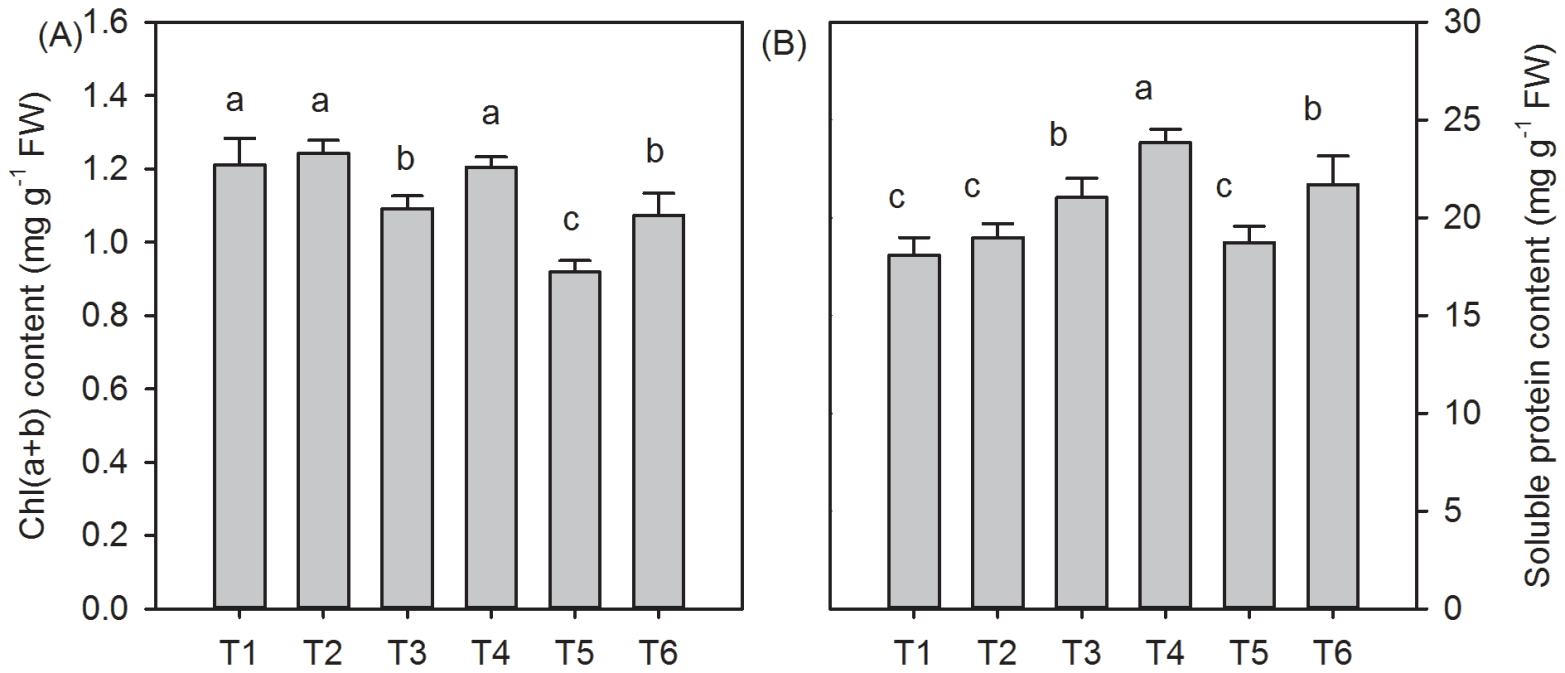
Effects of SA on the chl (a+b) (A) and soluble protein contents (B) in *T. grandis* grown under salt stress conditions (means ± SD). Treatments: T1, distilled water without SA; T2, distilled water with 0.5 mmol SA; T3, 0.2% NaCl without SA; T4, 0.2% NaCl with 0.5 mmol SA; T5, 0.4% NaCl without SA; and T6, 0.4% NaCl with 0.5 mmol SA. Different letters indicate significant differences (*P*<0.05) according to an LSD test; the same letter indicates no significant differences between the treatments, n = 5.

Chlorophyll is a biologically important pigment that is used for the photosynthetic conversion of inorganic molecules or ions into organic bio-molecules. Salt stress caused a decrease in the chl(a+b) content compared with that of the non-salt-treated seedlings. The contents of chl(a+b) decreased by 9.9% under 0.2% saline conditions and 24.1% under 0.4% saline conditions app:addword:respectively (Fig. 3A). The foliar spray of SA under salt stress conditions significantly reduced the decreases in the contents of chl(a+b) by approximately 10% (*P*<0.05) under 0.2% saline conditions and 17% (*P*<0.05) under 0.4% saline conditions compared that of the non SA-treated plants (Fig. 3A). In contrast to the change in the chlorophyll contents under saline condition, the salt stress increased the total soluble protein content. The 0.2% NaCl treatment significantly increased the soluble protein content (*P*<0.05); however, no obvious increase was found between the 0.4% NaCl- and non NaCl-stressed plants (*P*>0.05) (Fig. 3B). The addition of the SA solution significantly increased the soluble protein contents under NaCl conditions, and the soluble protein content in the SA-treated plants increased by 13.3% (*P*<0.05) under 0.2% saline conditions and 15.9% (*P*<0.05) under 0.4% saline conditions (Fig. 3B). However, the SA treatment did not affect the contents of chl(a+b) and soluble protein compared with those of the SA- and distilled water-treated seedlings (*P*>0.05).

### Activities of antioxidative enzymes and cellular membrane damage of *T. grandis* under different salt stress and SA treatments

**Figure 4:**
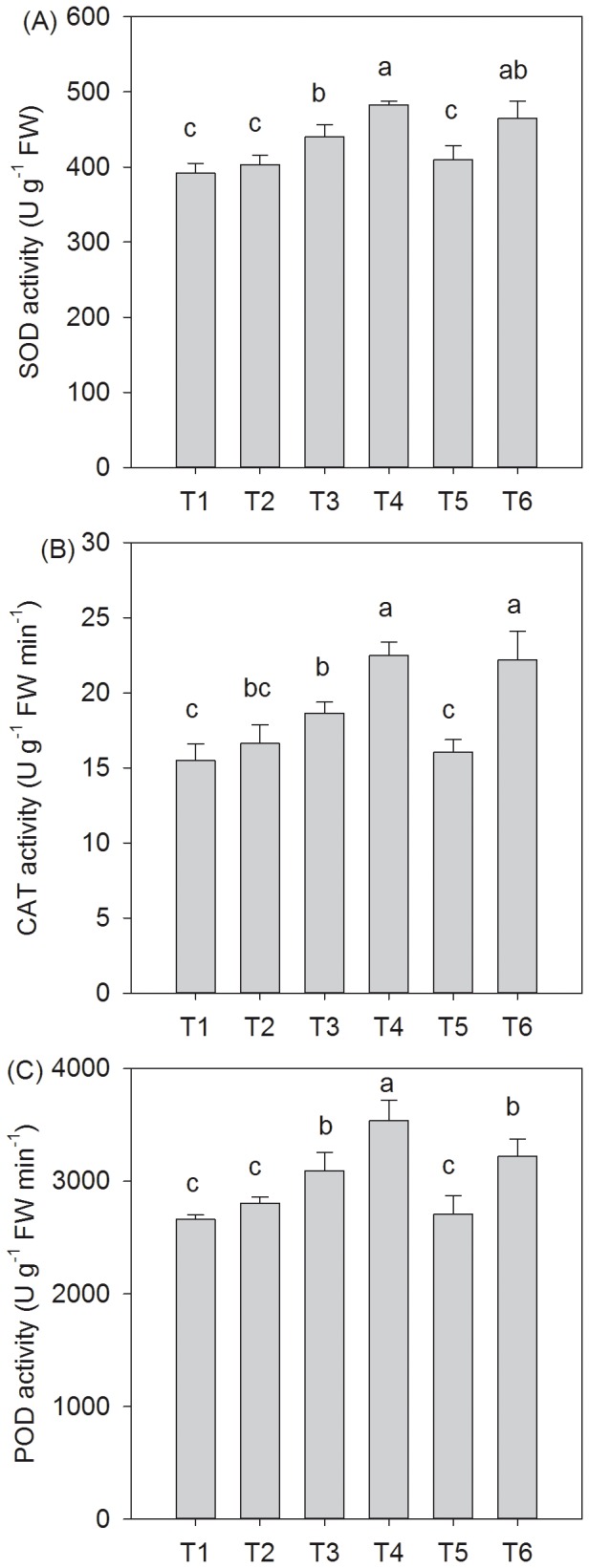
Influence of the SA treatments on the SOD (A), CAT (B), and POD (C) activities in *T. grandis* grown under salt stress conditions. Treatments: T1, distilled water without SA; T2, distilled water with 0.5 mmol SA; T3, 0.2% NaCl without SA; T4, 0.2% NaCl with 0.5 mmol SA; T5, 0.4% NaCl without SA; and T6, 0.4% NaCl with 0.5 mmol SA. Different letters indicate significant differences (*P<0.05*) according to an LSD test, n = 5.

The activities of SOD, CAT, and POD in *T. grandis* seedlings were significantly affected by the salt stress and SA treatments. With the increasing extent of salt stress, the activities of SOD, CAT, and POD increased markedly and then significantly decreased (Fig. 4). The SA treatment significantly enhanced the activities of SOD, CAT, and POD under salt stress, and the increases in the activities of SOD, CAT, and POD were approximately 9.8%, 20.6%, and 14.4% under 0.2% NaCl conditions and 13.4%, 38.2%, and 18.9% at 0.4% under NaCl conditions, respectively (Fig. 4).

**Figure 5:**
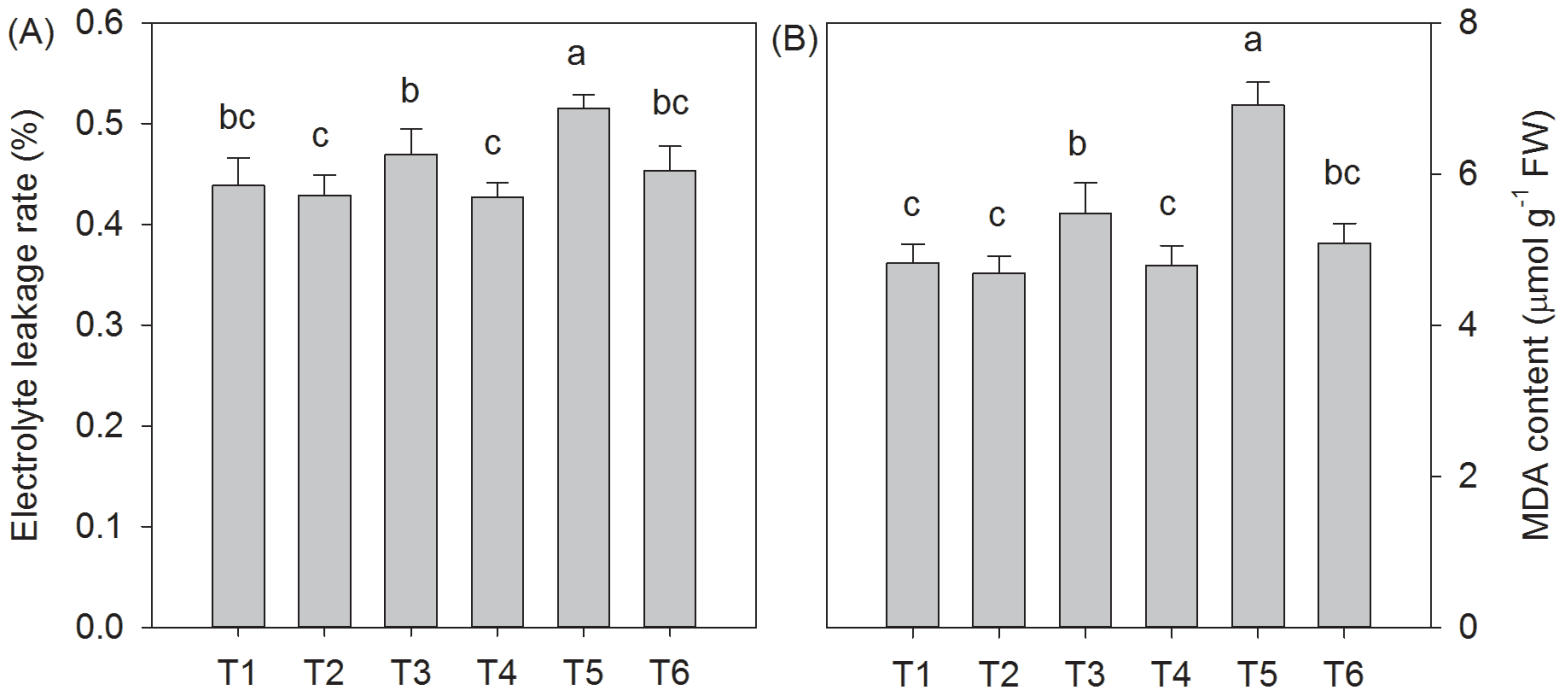
Effects of the SA treatments on the electrolyte leakage rate (A) and MDA contents (B) in *T. grandis* seedlings grown under salt stress conditions. Treatments: T1, distilled water without SA; T2, distilled water with 0.5 mmol SA; T3, 0.2% NaCl without SA; T4, 0.2% NaCl with 0.5 mmol SA; T5, 0.4% NaCl without SA; and T6, 0.4% NaCl with 0.5 mmol SA. Different letters indicate significant differences (*P*<0.05) according to an LSD test, n = 5.

Salt stress caused membrane injury in the leaves of *T. grandis* seedlings (Fig. 5). The relative electrolyte leakage rate (REC) increased by 6.9% and 17.4% under 0.2% and 0.4% NaCl conditions, respectively, and the MDA content increased by 13.5% and 43.3% under 0.2% and 0.4% NaCl conditions, respectively. The SA treatments prevented lipid peroxidation and then alleviated the membrane injury of *T. grandis* seedlings. Compared with the non-SA-treated plants, the reductions in the REC and MDA content in the SA-treated seedlings were approximately 9.0% (*P*<0.05) and 12.6% (*P*<0.05) under 0.2% NaCl conditions and 12.0% (*P*<0.05) and 26.4% (*P*<0.05) under 0.4% NaCl conditions, respectively. Interestingly, no significant difference was found between the REC and MDA content between the SA- and distilled water-treated seedlings (*P*>0.05).

### Relationships between antioxidant enzymatic activities and cellular membrane damage

**Figure 6:**
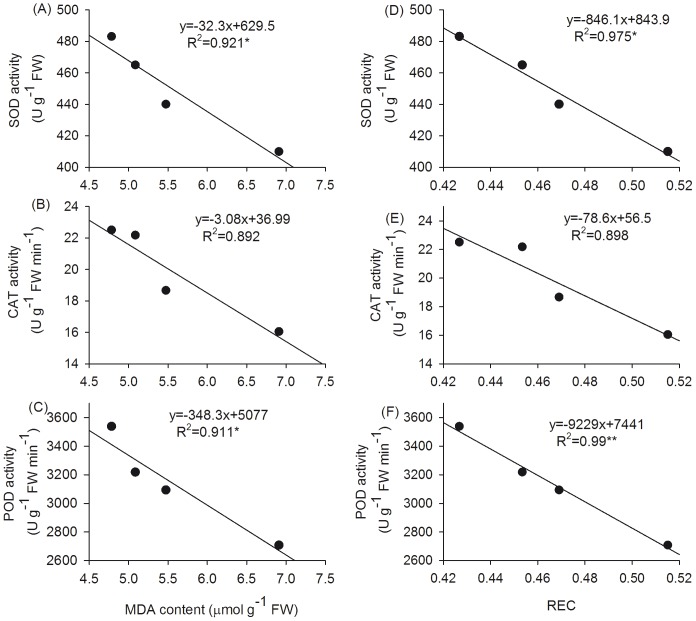
Relationship between MDA content and activities of SOD (A), CAT (B) and POD (C), and between REC and activities of SOD (D), CAT (E) and POD (F), respectively in *T. grandis* seedlings grown under 0.2% and 0.4% salt stress conditions. Each point represents the mean of 5 seedlings. *and **Significant at *P*<0.05 and *P*<0.01, respectively.

MDA content shows linear and negative correlation with SOD and POD activities (Fig. 6A, C, *P*<0.05) likewise, REC content also shows negative correlation with SOD and POD activities (Fig. 6D, F, *P*<0.05). However, there were no significant negative relationships between MDA content and CAT activity, and also between membrane permeability and CAT activity, respectively (Fig. 6B, E, *P*>0.05).

## Discussion

Salt stress constrains plant growth by adversely affecting various physiological and biochemical processes, such as photosynthesis, antioxidant phenomena, proline metabolism, and osmolyte accumulation [30], [31], [32], [33]. Given that SA plays key roles in the regulation of plant growth, development, the interaction with other organisms, and the responses to environmental stresses [34], [35]. However, there is some controversy in the literature regarding whether SA can counteract the adverse effects of salt stress [10], [30]. In this study, a marked reduction in the growth of both the shoots and roots of *T. grandis* seedlings was observed under increasing salt stress (Fig. 1). These studies show that SA treatment recovered the growth rate of *T. grandis* seedlings from high salt conditions (0.4% NaCl), when compared with their corresponding non-SA applied plants (Table 1). This is in consistent with the reports of Idrees et al. [31] where it was shown that SA treatment ameliorated the adverse effects of salt stress in terms of growth parameters in *Catharanthus roseus*. Coronado et al. [36] also showed that applying aqueous solutions of SA as a spray solution to the shoots of soybean significantly increase the growth of the shoots and roots under greenhouse or field conditions. Interestingly, no significant differences were found in the dry mass of the shoots and roots between the plants with and without SA treatment under 0% NaCl stress conditions (Table 1), suggesting that exogenous SA did not positively affect the growth of *T. grandis* seedlings that were grown under non-saline and low-saline conditions. This result was not consistent with the report of Kaydan et al. [37], who found that a pre-sowing soaking treatment of the seeds that were treated with SA positively affected the shoot and root dry mass in wheat seedlings under both saline and non-saline conditions.

Growing leaf cells can be maintained through the accumulation of solutes. Proline accumulation is one of the most frequently reported adjustments that is induced by salt stress in plants and is often considered to be involved in maintaining the water content. Increases in the leaf RWC and accumulation of free proline in *T. grandis* seedlings after 30 days of salt stress under SA treatment might be an adaptive feature in improving its succulence and maintaining the water balance in response to salinity-induced osmotic stress (Fig. 2). A similar result was reported by Misra and Saxena [32], who found that SA alleviated the salt stress of lentil by improving the proline metabolizing system. Moreover, proline enhances the salt-tolerance of *Pancratium maritimum* L. by up-regulating stress-protective proteins [38]. The application of SA induced an increase in the protein content under both saline stress conditions (Fig. 3B). Therefore, it has been concluded that SA ameliorates the stress generated by NaCl through proline accumulation, which was involved in the synthesis of protective proteins that are necessary for the stress response. However, to test this hypothesis, further studies are necessary to determine which stress-protective protein would increase under a stress environment.

Stomatal closure is one of the earliest responses to salt stress. Compared to the non-salt-treated *T. grandis* seedlings, both the Pn and Gs in *T. grandis* seedlings under 0.2% NaCl conditions decreased by approximately 16% and 39%, respectively, suggesting that the decrease in the photosynthetic rate was associated with the closure of stomata under 0.2% NaCl stress. SA alleviated the negative effects of 0.2% NaCl stress on leaf photosynthesis by increasing Pn and Gs. Fariduddin et al. [33] also found that the exogenous application of SA could enhance the net photosynthetic rate, stomatal conductance, and transpiration rate in *B. juncea*. However, non-stomatal factors, such as enzyme-related activities at the chloroplast level, the efficiency of PSII, the concentration and activity of the Rubisco enzyme, and the supply of ATP and NADPH to the photosynthetic carbon reduction cycle might also affect the Pn in the leaves under stressful conditions [39]. Furthermore, Brugnoli and Bjökman [40] noted that both stomatal and non-stomatal limitations accounted for a reduction in photosynthesis. In this study, compared with the 0.2% NaCl treatment, the Pn further decreased, although the Gs did not obviously change in *T. grandis* seedlings under 0.4% NaCl conditions, suggesting that other non-stomatal limiting factors might affect photosynthesis under 0.4% NaCl conditions. The enhancement of the effects of SA on the Pn was attributable to its stimulatory effects on the pigment contents and Rubisco enzyme activity [41]. In this study, the SA-treated seedling leaves exhibited higher Pn and lower Ci contents, consistent with higher levels of Gs, chlorophyll, and soluble proteins than those of the 0.4% NaCl-treated plants that were treated with SA (Table 2; Fig. 3A,B). These results indicate that the application of SA induced the severely salt-stressed *T. grandis* seedlings to effectively use the available CO_2_ inside the leaves and increased the Pn by up-regulating the related photosynthetic enzyme activities at the chloroplast level.

The photosynthetic electron transport system is a major source of ROS in plant tissues and has the potential to generate singlet oxygen ^1^O_2_ and superoxide (O_2_.^−^). Consequently, the accumulation of reduced intermediates in the photosynthetic electron transport chain results in the formation of ROS, thereby damaging the membrane system and photosynthetic complexes [42]. Plants can activate antioxidative defense systems to protect themselves from the harmful effects of salinity-induced oxidative stress. In this study, salinity stress induced drastic changes in the activities of antioxidative enzymes, including SOD, CAT, and POD. There were dramatic increases in the activities of SOD, CAT, and POD under 0.2% salt stress conditions, although there was only a slight increase under 0.4% salt stress conditions compared to the distilled water-treated seedlings (Fig. 3), suggesting that with increasing salt stress, the ability of *T. grandis* seedlings to resist salt stress decreased. SOD and CAT, which act synergistically to resist oxidative damage induced by salt stress in *T. grandis* seedlings, were also observed in other plant tissues under NaCl stress [43]. After the application of exogenous SA, the plants showed higher activities of SOD, CAT, and POD than those of the non-SA-treated plants grown under 0.2% and 0.4% salt stress conditions (Fig. 4). Similarly, SA enhanced the activities of CAT, POD, and SOD when sprayed exogenously on salinity-stressed *B. juncea* plants [44].

One of the major injuries due to salinity stress is the damage of plant cell membranes, which can impair their ion selectivity. This deficiency results in the exosmosis of intracellular ions and organic matter and the infiltration of extracellular toxic ions, ultimately disrupting plant physiological and biochemical processes. The maintenance of cellular membrane integrity under salt stress is considered to be an integral part of the salinity tolerance mechanism [45]. The membrane damage caused by different abiotic stresses, including salinity, is largely mediated through the end product of lipid peroxidation (MDA) [46]. In this study, a significant positive correlation (r = 0.98, p = 0.0029) was observed between the MDA concentration and membrane permeability. An increase in the electrical conductivity with increasing salt stress indicated an elevated leakiness of ions due to a loss of membrane integrity (Fig. 4), which is in agreement with results described by Parida and Das [47]. The application of exogenous SA could ameliorate the membrane deterioration in plants under salt stress and facilitate the maintenance of membrane functions [7], [45]. We also found that the SA treatment significantly reduced the increase in the electrolyte leakage rate and MDA content in *T. grandis* seedlings under salt stress (Fig. 5).

The photosynthetic pigment chlorophyll plays an important role in light absorption and energy transduction and is essential for photosynthesis. The reduction of leaf chlorophyll under high salinity has been attributed to the destruction of pigments and the instability of the pigment-protein complex [48]. In this study, NaCl treatment reduced the chlorophyll content of *T. grandis* seedlings, and there was a significantly positive and linear relationship between Pn and the chlorophyll content (r = 0.96, p = 0.0099) (Fig. 3A). The addition of SA to NaCl-stressed plants markedly increased the chlorophyll content, suggesting that the increase in chloroplast pigments under the SA treatment might be due to the ability of SA to increase the activity of certain enzymes, thereby stimulating chlorophyll biosynthesis or reducing chlorophyll degradation, leading to increased net photosynthesis under salt stress tolerance. The reduction in chlorophyll due to osmotic stress has been ascribed to the strong damage to chloroplast membranes, which increases the membrane permeability or loss of membrane integrity [49]. Indeed, a strong negative correlation between the chlorophyll and MDA contents (r = 0.99, *p* = 0.001) was observed in this study. Membrane oxidative damage can be prevented by antioxidative enzymes. Correlation studies were demonstrated for MDA content and REC content against the enzymatic activities of SOD, CAT and POD in *T. grandis* seedlings grown under 0.2% and 0.4% salt stress conditions. We found that both MDA content and membrane permeability shows significant negative correlation against the activities of SOD and POD (Fig. 6A,C,D,F, *P*<0.05), while there is no significant negative correlation between MDA content and CAT activity, and also between membrane permeability and CAT activity (Fig. 6B,E, *P*>0.05). Therefore, the observed increase in the growth and CO_2_ assimilation under salt stress and SA treatment might be due to the enhanced activities of SOD and POD, which protect membrane integrity, leading to an increase in the amount of photosynthesis pigments and net photosynthesis rate.

In conclusion, our study showed that salt stress decrease the vegetative growth and CO_2_ assimilation in *T.grandis* together with increased membrane damage and lipid peroxidation. SA efficiently ameliorated the negative effects of salt stress over the growth and photosynthesis of *T. grandis* by increasing the chlorophyll content and also enhancing the plants antioxidant enzyme mechanisms, thus alleviate the membrane oxidative damage from salinity. To our knowledge, this is the first report of salt tolerance mechanisms in *T. grandis* seedlings. The results of this study can also provide a useful guide for the conservation and large-scale plantation of *T. grandis*.
